# Investigating the Effect of Active Site Density in Transition Metal-Doped Graphene on CO Gas Sensing Performance: A DFT Study

**DOI:** 10.3390/s26072128

**Published:** 2026-03-30

**Authors:** Siyu Wang, Yahui Li, Tao Zhou, Panagiotis Tsiakaras

**Affiliations:** 1 School of Physical Science and Technology, Guangxi University, Nanning 530004, China; 2307301138@st.gxu.edu.cn; 2 Laboratory of Alternative Energy Conversion Systems, Department of Mechanical Engineering, School of Engineering, University of Thessaly, Pedion Areos, 38834 Volos, Greece; 3 College of Environmental Science and Engineering, Tongji University, Shanghai 200092, China

**Keywords:** CO gas sensing, density functional theory, active site density, bimetallic doping, spin polarization, graphene

## Abstract

**Highlights:**

**What are the main findings?**

- Active-site density non-monotonically affects the electronic structure of FePt–N4–C.
- CO chemisorbs on Fe sites, but only physisorbs on Pt sites.
- Heteronuclear FePt sites show stronger CO adsorption than homonuclear PtPt sites.
- Spin polarization changes the density-dependent trend of CO adsorption.

**What are the implications of the main findings?**

- FePt–N4–C is a promising platform for tunable CO capture and sensing.
- Active-site design, spin state, and temperature can be used to regulate CO adsorption behavior.

**Abstract:**

Developing sensitive and reversible CO sensors requires precise control of material–analyte interactions. Using DFT, we investigate CO sensing on bimetallic (Fe, Pt) anchored on N-doped graphene (TM_2_–N_4_–C), focusing on active-site density effects. Three densities are considered: low (12.7 Å), medium (8.5 Å), and high (4.2 Å). FePt–N_4_–C band gaps exhibit non-monotonic tuning, approaching metallicity at high density. CO chemisorbs on Fe sites, but physisorbs on Pt sites. FePt exhibits stronger synergistic adsorption than homonuclear counterparts. While adsorption generally strengthens with density, spin-polarized calculations qualitatively reorder this trend via spin delocalization. High temperatures drastically improve recovery; low-density FePt–N_4_–C reaches 65 s at 498 K. Three design principles emerge: low-density heteronuclear systems for reversible sensing, medium-density high-spin states for ultra-sensitive capture, and high-density configurations for work function sensors. This work establishes active site density as a key electronic and kinetic knob for graphene-based CO sensors.

## 1. Introduction

The escalating emissions of toxic gases from fossil fuel combustion, industrial processes, and accidental leaks present a severe threat to both ecological systems and public health, thereby challenging the global transition to sustainable energy and industrial development [1,2,3,4]. Beyond the primary atmospheric components, sulfide, carbide, and nitride gases can cause multiple adverse health effects even at trace concentrations, including respiratory damage, neurological impairment, and even fatal risks [5,6,7]. One of the gases which have been extensively studied is CO gas because it is known to be extremely harmful to the human body, and it is also a main cause of air pollution. The CO is odorless, colorless, nonirritant, and tasteless, which means it is difficult to detect by an exposed person. However, CO is a relatively stable molecule and interacts weakly with many conventional sensors; traditional sensors have slow response speeds, low sensitivity, and it is difficult to achieve accurate and rapid detection. It is crucial to develop sensing materials and technologies with high sensitivity and selectivity for carbon monoxide.

Gas sensing plays a pivotal role in environmental monitoring and safety, with the performance of such devices fundamentally dictated by their core sensing materials [8,9,10,11]. During the past decade, two-dimensional (2D) nanomaterials have become a research hotspot due to their unique physicochemical properties, such as extremely high specific surface area and tunable electronic structures. In two-dimensional materials, graphene is attractive due to its high surface area and excellent electrical conductivity, which are beneficial for surface-adsorption-driven sensing [12,13,14,15,16]. For instance, pioneering theoretical studies by Lee et al. [17] and Leenaerts et al. [18] have systematically revealed the adsorption behaviors of various organic and inorganic molecules on graphene surfaces using density functional theory (DFT). While promising, pure graphene still faces many practical limitations in gas sensing. Its inherent chemical inertness leads to weak interactions with many critical gas molecules, such as CO, resulting in low sensitivity and poor selectivity [19]. This intrinsic limitation has spurred extensive research into performance enhancement strategies, primarily focusing on material modification, structural design, and the development of composite systems [20,21,22].

By doping with transition metal (TM) atoms, a large number of active sites can be introduced and their electronic structure can be significantly modulated, thereby overcoming the inertness of graphene and ultimately enhancing its adsorption and sensing response to target gases [23,24,25,26,27]. Meanwhile, the coordination of nitrogen atoms forms transition metal–nitrogen (TM–N_x_) configurations, which effectively suppress the agglomeration of metal atoms [28], thereby supporting the preparation of sensor materials with enhanced stability [29,30]. Lee et al. [31] investigated the use of bubble transfer method to transfer graphene onto SiO_2_-covered silicon substrates to obtain graphene materials with different metal loads. They evaluated the effect of MO_x_ nanostructures with position selectivity and density control in hydrogen sensor applications by controlling the position of MO_x_ nanostructures and growth parameters such as nucleation density and growth rate. Michela Cittadini et al. [32] prepared optical gas sensors by depositing graphene oxide sheets on single-layer gold nanoparticles and causing optical changes when exposed to different gases through the coupling between the sheets and the nanoparticles. Due to the electron transfer between gold nanoparticles and the sp^2^ hybridized carbon two-dimensional sheet of graphene oxide, the surface plasma resonance band moves in the presence of reducing gas and oxidizing gas, thus demonstrating good gas sensitivity. Meanwhile, the doping of non-metallic elements can also regulate the behavior of adsorbed gases by constructing specific adsorption channels. Kwon et al. [33] achieved an average detection limit of 0.83 ppm for oxidizing gases such as NO_2_ or SO_2_ by enhancing the electrostatic interaction between graphene-rich and electron-deficient NO_2_ through the use of N-doped graphene with vinyl amine, thereby improving response, recovery and long-term sensing stability, and strengthening current drop through the N-channel. The local microenvironment of a catalyst, which is critically regulated by the density of adsorption sites, exerts a profound influence on both its selectivity and activity [34]. To illustrate this, Shan et al. [35] demonstrated that the optimal hydrogen evolution reaction activity was achieved when the distance between the Fe–N_4_ and Mn–N_4_ active sites was approximately 8.65 Å, while Jin et al. [36] identified an optimal distance of approximately 7 Å for the oxygen reduction reaction. Despite these advances, the effect of intersite interactions on the adsorption of gases remains an area that has not been fully explored.

Furthermore, for Fe-containing systems, spin polarization introduces an additional degree of freedom in modulating gas–surface interactions. In nitrogen coordination environment, Fe centers can exhibit multiple spin states (S = 0, 1, 2) [37]. The resulting spin polarized electronic structure can significantly influence chemisorption energetics and magnetic properties [38,39,40,41]. However, despite these advances in understanding intersite interactions and spin effects in catalysis, their combined influence on gas adsorption and sensing mechanisms—particularly how site density modulates spin-dependent behavior—remains a relatively unexplored frontier.

In this work, the CO gas sensing performance of TM_2_–N_4_–C (TM = Fe and Pt) with different active site densities is systematically investigated by DFT calculations. The structural stability is evaluated through the formation energy (*E*_f_). The structural and electronic properties, along with CO adsorption kinetics, of TM_2_–N_4_–C systems are analyzed to unravel how active-site density controls the trade-off between strong binding and feasible desorption via charge transfer and spin polarization. The synergistic effect of active site density and spin polarization was also investigated. Furthermore, the recovery time is calculated to evaluate the desorption capability of CO gas molecule from the sensing material surface. Through this multifaceted investigation, we aim to establish clear design principles for engineering graphene-based sensing materials with optimized active site density, composition, and spin state for high-performance and tunable CO detection.

## 2. Computational Details

This study employed the DMol^3^ code within the Materials Studio 2020 software package for all spin-polarized DFT calculations [42,43]. The exchange-correlation effects were described by the Perdew–Burke–Ernzerhof (PBE) functional under the generalized gradient approximation (GGA) framework [44]. We utilized an all-electron double numerical plus polarization (DNP) basis set, which is comparable in quality to the Gaussian 6-31G(d,p) basis set and offers good accuracy for geometric and electronic structure calculations of systems containing transition metals. The Brillouin zone was sampled using a 3 × 3 × 1 Monkhorst–Pack k-point grid for the structural relaxations and subsequent property calculations of the modeled 2D periodic slabs. The self-consistent field (SCF) convergence threshold was set to 2 × 10^−5^ Ha per atom. Geometry optimizations were considered converged when the energy change, maximum force, and maximum displacement were less than 2 × 10^−5^ Ha, 0.004 Ha/Å, and 0.005 Å, respectively. To eliminate spurious interactions between periodic images in the vertical direction, a vacuum layer of 15 Å was applied along the *z*-axis, which is sufficient for our slab models.

To estimate the thermodynamic stability of the designed TM_2_–N_4_–C models, the formation energy per metal atom (*E*_f_) was calculated using the following equation:

*E*_f_ = (*E*_total_ + *n*_C_*μ*_C_ – *E*_graphene_ − *n*_N_*μ*_N_ − *n*_TM_*μ*_TM_)/*n*_TM_ (1)

where the *E*_total_ and *E*_graphene_ represent the total energies of TM_2_–N_4_–C and graphene, respectively. The *n*_C_, *n*_N_, and *n*_TM_ denote the number of C atoms removed during the construction of TM_2_–N_4_–C, as well as the number of N and TM atoms added. The chemical potential *μ*_C_, *μ*_N_, and *μ*_TM_ are referenced to the energy per atom in graphene, half the energy of an N_2_ molecule in the gas phase, and the energy per atom in the bulk metal crystal (for Fe and Pt), respectively.

The adsorption energy ($E_{\mathrm{ads}}$) of a CO molecule on the substrate was calculated as:

*E*_ads_ = *E*_total_ − *E*_gas_ − *E*_substrate_ (2)

where *E*_total_ refers to the total energy of the substrate with the gas molecule adsorbed. *E*_substrate_ corresponds to the energy of the clean substrate. *E*_gas_ is the energy of the free gas molecule.

The recovery time (*τ*) is defined as the duration required for the sensor to return to its initial state after the complete removal of the gas [45,46]. It is calculated using the following equation:

*τ* = *v*^−1^ *e*^−*E*ads/*K*B*T*^ (3)

where *v* represents the attempt frequency (taken as 10^12^ s^−1^, a typical value for surface processes), *K*_B_ denotes the Boltzmann constant (8.62 × 10^−5^ eV/K), and *T* is the temperature in Kelvin, and *E*_ads_ is the adsorption energy defined in Equation (2). It should be noted that this formula provides a first-order estimate, assuming the adsorption energy barrier for desorption is approximately equal to ∣$E_{\mathrm{ads}}$∣, and does not explicitly account for the entropic contribution. The recovery times were evaluated at three representative temperatures: 298 K (room temperature), 398 K, and 498 K, to assess the temperature-dependent desorption behavior.

Spin-polarized (spin-unrestricted) DFT calculations were performed, with all other settings unchanged, to evaluate spin-state effects on adsorption in Fe-containing systems. Following previous studies [37], the non-spin-polarized (S = 0) case was used as a nonmagnetic reference, and S = 1 and S = 2 were considered for the Fe center in FePt–N_4_–C. For S = 1 and S = 2, the multiplicities of these states are set to 3 and 5, respectively. The thermodynamically preferred spin state was identified by total-energy comparison (Table A1). Spin density and spin-resolved PDOS were analyzed to reveal the electronic origin of spin-dependent adsorption (Figure A2).

## 3. Results and Discussion

### 3.1. Model and Stability of the TM_2_–N_4_–C

**Figure 1:**
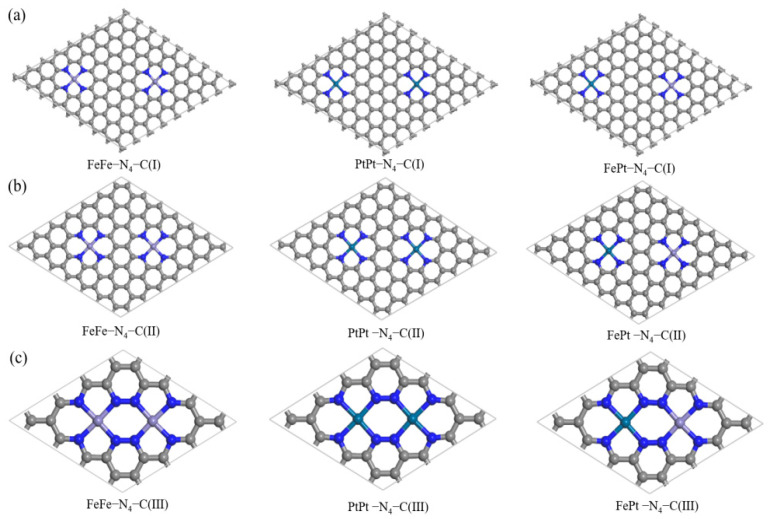
Optimized geometric structures of the three representative TM_2_–N_4_–C models with different active-site densities: (**a**) type I (low density, d ≈ 12.7 Å), (**b**) type II (medium density, d ≈ 8.5 Å), and (**c**) type III (high density, d ≈ 4.2 Å). Carbon, nitrogen, iron, and platinum atoms are depicted as gray, blue, purple, and cyan spheres, respectively.

To investigate the effect of active site density on CO gas adsorption, three models with bimetallic sites anchored on nitrogen-doped graphene were constructed based on previously reported experimental data [36,47,48], denoted as TM_2_–N_4_–C (TM = Fe and Pt). As illustrated in Figure 1, the center-to-center distance between adjacent bimetallic sites was precisely controlled at approximately 12.7 Å, 8.5 Å, and 4.2 Å for configurations I (low density), II (medium density), and III (high density), respectively. For each density, we investigated both homonuclear (FeFe, PtPt) and heteronuclear (FePt) metal pairs. DFT geometry optimization confirms that all TM_2_–N_4_–C structures retain a planar configuration, with the metal atoms firmly embedded in the graphene lattice without significant out-of-plane distortion, indicating their structural feasibility.

**Figure 2:**
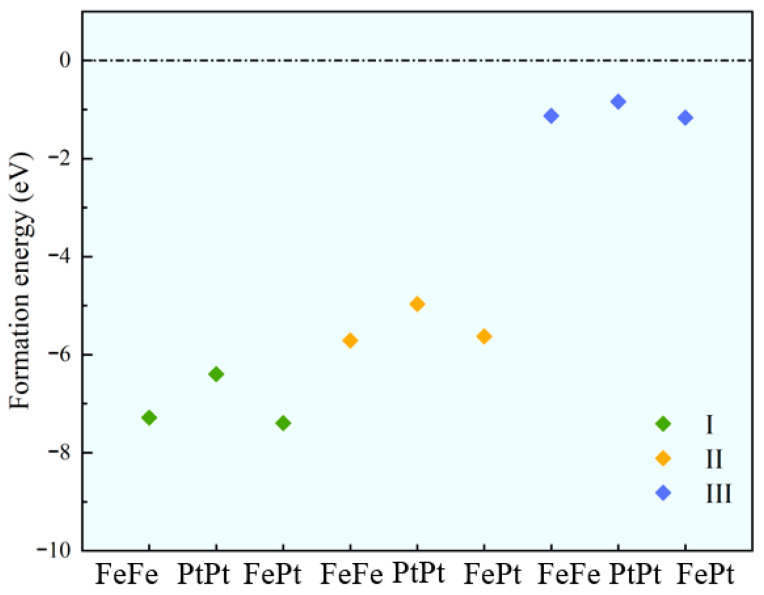
Formation energy of all TM_2_–N_4_–C configurations (TM = Fe, Pt). For a given metal composition, *E*_f_ becomes more negative as the active site density decreases (from type III to I). At a fixed site density, heteronuclear FePt systems consistently exhibit more negative formation energies than their homonuclear (FeFe, PtPt) counterparts, indicating superior thermodynamic stability.

The stability of a material represents a fundamental requirement for its practical deployment in sensor applications. We calculate the *E*_f_ of all TM_2_–N_4_–C configurations and plot the results in Figure 2. According to the criterion, a material with a negative *E*_f_ value (*E*_f_ < 0) is thermodynamically stable.

The results indicate that all calculated structures have negative *E*_f_ values, confirming their thermodynamic stability. Further analysis revealed that as the density of active sites decreases, the *E*_f_ value becomes more negative, indicating that the sensor becomes more stable. In addition, compared with the homonuclear metal atom-doped system, the material doped with heteronuclear metal atoms has a more negative formation energy, indicating that the heteronuclear doping strategy can effectively improve the overall stability of the material. Among them, FePt–N_4_–C(I) has the most negative formation energy of −7.39 eV, demonstrating the best thermodynamic stability. Furthermore, to further verify the rationality of the N_4_ coordination environment, we take the medium-density configuration as an example and construct FePt–N_3_–C(II) and FePt–N_5_–C(II) coordination structures for comparison. Their formation energies and CO adsorption energies are calculated. The formation energy of FePt–N_3_–C(II) is positive, indicating that this structure is thermodynamically unstable. Thus, the subsequent CO adsorption calculation on FePt–N_3_–C(II) is not considered. In contrast, the formation energy of FePt–N_5_–C(II) is −2.29 eV, suggesting its good structural stability. Notably, FePt–N_4_–C(II) presents a more negative formation energy than FePt–N_5_–C(II), demonstrating its higher thermodynamic stability.

**Figure 3:**
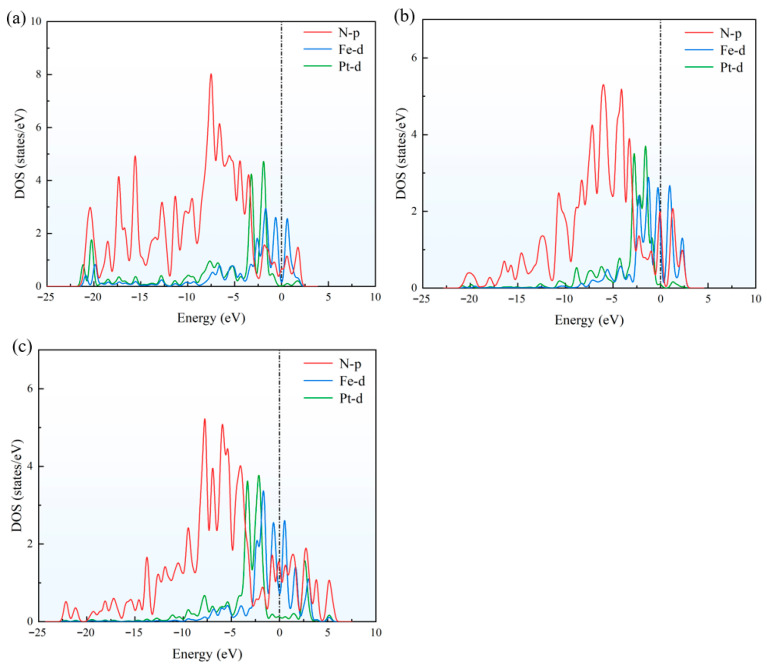
Density of states (DOS) of (**a**) FePt–N_4_–C(I), (**b**) FePt–N_4_–C(II), and (**c**) FePt–N_4_–C(III). The pronounced overlap between N-p and TM-d orbitals across a broad energy range confirms strong covalent TM–N bonding. The low-density configuration (I) exhibits the most extensive and intense hybridization features, correlating with its most negative formation energy.

In order to further analyze the interaction between the metal atom and the N-doped graphene substrate, the density of states (DOS) of TM_2_–N_4_–C was calculated and shown in Figure 3a–c.

The significant overlap and strong hybridization between the p orbital of N and d orbital of TM lead to the formation of stable TM–N bonds, which are crucial for the stable anchoring of TM. Analysis found that for FePt–N_4_–C(I), orbital hybridization is most widely distributed within the energy range, with obvious orbital overlap peaks in the energy range of −22 eV to 3 eV, indicating the highest orbital hybridization intensity and strongest interactions. This conclusion is mutually consistent with the most negative *E*_f_ calculated.

### 3.2. CO Gas Molecule Adsorbed on TM_2_–N_4_–C

The geometric optimization was conducted on an isolated CO molecule before the calculation of adsorption models, according to which the length of C–O is 1.14 Å, which is in excellent agreement with the values reported in previous studies [49,50,51].

**Figure 4:**
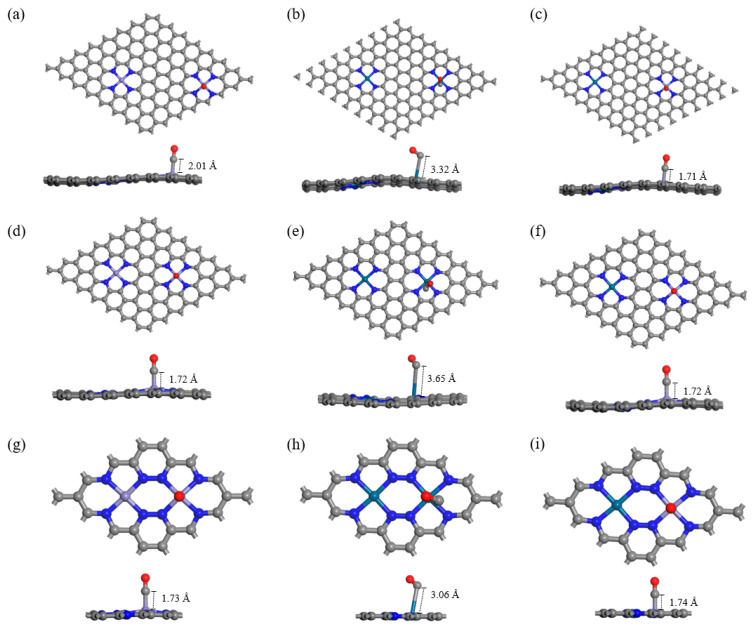
Optimal adsorption structures of CO adsorbed on (**a**) FeFe–N_4_–C(I), (**b**) PtPt–N_4_–C(I), (**c**) FePt–N_4_–C(I), (**d**) FeFe–N_4_–C(II), (**e**) PtPt–N_4_–C(II), (**f**) FePt–N_4_–C(II), (**g**) FeFe–N_4_–C(III), (**h**) PtPt–N_4_–C(III), and (**i**) FePt–N_4_–C(III). On Fe sites (FeFe and FePt), CO adopts a short, near-linear geometry (Fe–C–O angle ~175–180°), characteristic of chemisorption. On Pt sites (PtPt and the Pt site of FePt), CO binds with a long, tilted configuration (Pt–C–O angle ~108°), indicative of physisorption.

To determine the most stable adsorption configuration of CO, we systematically investigate two types of bonds (TM–C and TM–O), three CO adsorption angles (0°, 60°, and 90°), and two adsorption modes (side on and end on) on different active sites at each density. The CO is placed approximately 2 Å above the metal atoms. After structural optimization, the most stable configuration for TM_2_–N_4_–C to adsorb CO is obtained by comparing energy. Calculation results show that, regardless of the adsorption site, adsorption through the C atom is consistently more stable than through the O atom. Furthermore, in the PtFe system, CO adsorption on the Fe site exhibits a more negative adsorption energy than on the Pt site. Therefore, the Fe–C adsorption on the Fe site represents the most energetically favorable configuration. When CO molecules adsorb at the Fe site, regardless of the placement method, the adsorption distance remains stable at approximately 1.7 Å across different configurations (such as FeFe–N_4_–C and PtFe–N_4_–C) and varying active site densities, and CO molecules always maintain a spatial configuration almost perpendicular to the material surface. In contrast, when CO molecule adsorbs at Pt site, its behavior differs markedly from that at Fe site: the adsorption distance increases to approximately 3 Å, substantially greater than that at Fe site. Furthermore, CO molecules tilt at a certain angle with the material surface, forming a Pt–C–O bond angle of approximately 108°, as shown in Figure 4.

Subsequently, the adsorption energy of CO on TM_2_–N_4_–C was calculated, and the results are summarized in Table 1. The order of adsorption strength follows a clear density-dependent trend, with the most negative values observed on the high-density (III) configurations. Specifically, FePt–N_4_–C(III) exhibits the strongest adsorption (−1.52 eV), followed by FePt–N_4_–C(II) (−1.49 eV) and FeFe–N_4_–C(III) (−1.46 eV). The complete sequence is: FePt–N_4_–C(III) > FePt–N_4_–C(II) > FeFe–N_4_–C(III) > FeFe–N_4_–C(II) > FeFe–N_4_–C(I) > FePt–N_4_–C(I) > PtPt–N_4_–C(III) > PtPt–N_4_–C(II) > PtPt–N_4_–C(I), with corresponding adsorption energies of −1.52, −1.49, −1.46, −1.41, −1.37, −1.36, −0.28, −0.22, and −0.18 eV, respectively. The adsorption strength on the best-performing FePt–N_4_–C(III) (−1.52 eV) substantially surpasses that of many reported 2D sensing materials system, such as pristine graphene (−0.12 eV) [52], N-doped graphene (−0.40 eV) [52], BC_3_ [53], MoS_2_ (−0.13 eV) [54], CO/MnN_4_-Gra (−1.39 eV) [55], Au–Ti_3_C_2_ (−0.14 eV) [56], and G/ZnO heterojunction (−0.26 eV) [57]. For the stable FePt–N_5_–C(II) configuration, the adsorption energy of CO is calculated FePt–N_5_–C(II) is –1.02 eV, which is weaker than that of FePt–N_4_–C(II). Increasing the N coordination number may weaken the adsorption of CO.

Several key trends emerge from the data. (i) For the homonuclear FeFe system, the CO adsorption intensity increases monotonically with increasing site density: |*E*_ads_| increases from 1.37 eV (type I, low density) to 1.41 eV (type II, medium density) to 1.46 eV (type III, high density). The shortening of the Fe-Fe distance enhances the adsorption capacity of iron sites for carbon monoxide (CO). (ii) For heteronuclear FePt systems, the adsorption strength also increases with density, with values of −1.36 eV (I), −1.49 eV (II), and −1.52 eV (III). Notably, at each density, FePt shows comparable adsorption to FeFe at type I, but becomes stronger at types II–III, indicating density-enabled synergy. (iii) CO adsorption on Pt sites (in PtPt systems and the Pt site of FePt) is consistently weak (*E*_ads_ > −0.3 eV), characteristic of physisorption driven by van der Waals interactions. By contrast, CO is chemisorbed at Fe sites, involving significant electron transfer and orbital hybridization [58,59]. At a relatively large site spacing (approximately 12.7 Å), the adsorption energies of the two low-density sites, FePt–N_4_–C and FeFe–N_4_–C, are almost identical (–1.36 eV and −1.37 eV, respectively). This may be because the spacing minimizes the direct interaction between the two metal atoms, making the FePt dimer more similar to two isolated iron sites. These adsorption trends are consistent with the electronic structure analyses presented in the following sections, particularly the density-dependent upshift of the Fe d-band center.

**Figure 5:**
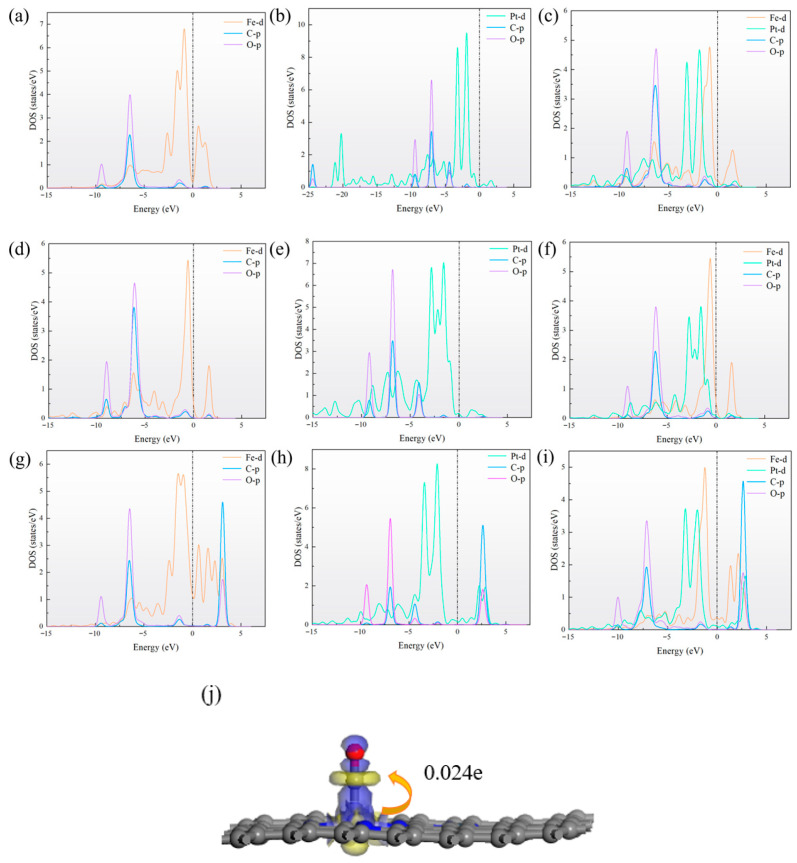
DOS of CO adsorbed on (**a**) FeFe–N_4_–C(I), (**b**) PtPt–N_4_–C(I), (**c**) FePt–N_4_–C(I), (**d**) FeFe–N_4_–C(II), (**e**) PtPt–N_4_–C(II), (**f**) FePt–N_4_–C(II), (**g**) FeFe–N_4_–C(III), (**h**) PtPt–N_4_–C(III), and (**i**) FePt–N_4_–C(III). (**j**) Deformation charge density of CO adsorbed on FePt–N_4_–C(II). The blue and yellow indicate electron accumulation and depletion, respectively. The iso-surface value is set as 0.05 e/Å^3^.

To gain deeper insight into the adsorption behavior of CO on the sensor material, we calculate the DOS of CO gas molecule adsorbed on TM_2_–N_4_–C, as shown in Figure 5. Obvious orbital overlap is observed between the d orbitals of TM and the p orbitals of the C and O atoms in CO, indicating the occurrence of orbital hybridization. It also confirms the existence of a certain interaction between them. Taking FePt–N_4_–C(II) as an example, we calculated Mulliken charge and deformation charge density. Mulliken charge analysis shows an electron transfer of 0.024 e to the CO molecule upon adsorption, identifying it as an electron acceptor on the FePt–N_4_–C(II) substrate. This is consistent with the results of DOS analysis.

**Figure A1:**
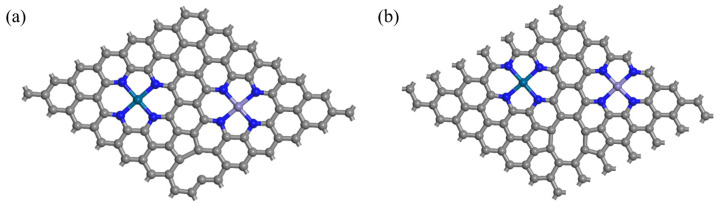
Optimal structures of (**a**) FePt–N_4_–C(II) (single vacancy), and (**b**) FePt–N_4_–C(II) (double vacancies).

**Figure A2:**
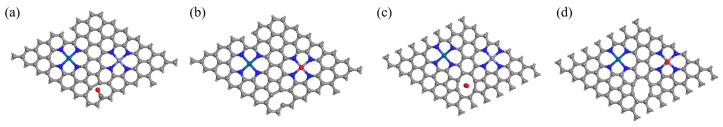
Optimal adsorption structures of CO adsorbed on the (**a**) defect sites and (**b**) Fe site of FePt–N_4_–C(II) with single vacancy. CO adsorbed on the (**c**) defect sites and (**d**) Fe site of FePt–N_4_–C(II) with double vacancies.

To investigate the impact of defects of graphene on the active sites, adsorption of CO of TM–N_4_–C, we choose FePt–N_4_–C(II) as an example and construct two types of defects on graphene, namely FePt–N_4_–C(II) (single vacancy) and FePt–N_4_–C(II) (double vacancies), respectively. The optimal configurations of them are displayed in Figure A1 and the structure of CO adsorption is illustrated in Figure A2. The Pt–N and Fe–N bond lengths remain almost unchanged (<0.05 Å) after introducing defects, indicating the high stability of the active site. The CO adsorption energies on the defect sites themselves are very weak, (FePt–N_4_–C(II) (single vacancy) with the adsorption energy of −0.28 eV and FePt–N_4_–C(II) (double vacancies) with the adsorption energy of −0.24 eV), much lower than that on the Fe site of FePt–N_4_–C(II) with single vacancy (−1.48 eV) and double vacancies (−1.54 eV). Moreover, the adsorption energy on FePt–N_4_–C(II) with defects changes less compared to the perfect system.

### 3.3. Electronic Structure Analysis

**Figure 6:**
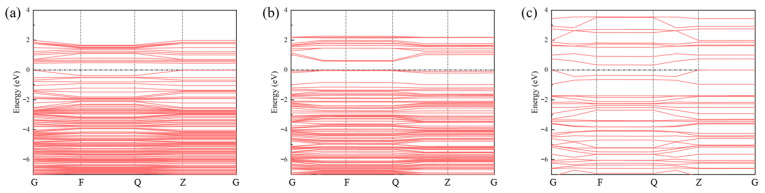
Band structures of (**a**) FePt–N_4_–C(I), (**b**) FePt–N_4_–C(II), and (**c**) FePt–N_4_–C(III). The Fermi level is set to 0 eV. The band gap values are 0.43 eV, 0.59 eV, and 0.019 eV, respectively, demonstrating a non-monotonic density-dependent modulation.

To elucidate how active-site density modulates the intrinsic electronic properties of the sensing material, we calculated the band structures of FePt–N_4_–C at three representative densities (Figure 6a–c). The band gap exhibits a non-monotonic evolution with increasing density: 0.43 eV (low density, type I) → 0.59 eV (medium density, type II) → 0.019 eV (high density, type III), approaching near-closure at the highest density.

This intriguing trend originates from a density-dependent transition in the dominant inter-site coupling regime. At low density (I, d ≈ 12.7 Å), the FePt dual sites are electronically isolated, behaving as independent impurity states within the graphene band gap. At medium density (II, d ≈ 8.5 Å), the reduced inter-site distance enables partial wavefunction overlap, leading to collective band formation and a downshift of the valence band maximum, which widens the gap. At high density (III, d ≈ 4.2 Å), the proximal FePt sites undergo strong direct d–d orbital hybridization, creating new dispersive bands that bridge the original gap and result in a nearly metallic state. This near-zero band gap implies significantly enhanced intrinsic conductivity, which is beneficial for facilitating charge transport in resistive-type gas sensors.

**Figure 7:**
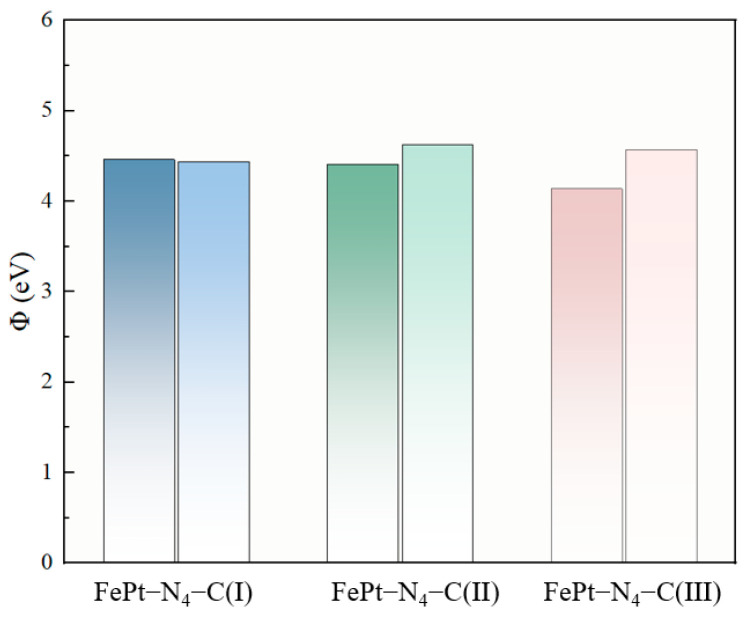
Calculated work functions of FePt–N_4_–C(I), FePt–N_4_–C(II), and FePt–N_4_–C(III) before and after CO adsorption. The ΔΦ values are −0.03, +0.21, and +0.44 eV, respectively. The high-density configuration (III) exhibits the most significant surface electronic response.

The work function (Φ), a critical physical parameter for characterizing the surface electronic states of materials, refers to the minimum energy necessary for electrons to break free from the material’s surface. In the context of gas sensing, the adsorption of gas molecules onto the material surface triggers the redistribution and transfer of surface charges, which in turn brings about a variation in the material’s work function. As this change exerts a direct regulatory effect on key electrical properties of the material, such as conductivity and electrode potential, monitoring the shift in work function has evolved into a central approach for assessing gas sensing performance. The calculated values of Φ of FePt–N_4_–C(I), FePt–N_4_–C(II), and FePt–N_4_–C(III) with and without CO molecule adsorption are plotted in Figure 7.

Strikingly, the high-density FePt–N_4_–C(III) configuration displays a ΔΦ more than three times larger than that of the other two densities. This distinct work function shift can be attributed not only to the strongest orbital hybridization, charge redistribution at high density, and the nearly closed band gap that facilitates more efficient charge transfer at the interface, but also to the enhanced surface dipole moment induced by CO molecules adsorbed at high density, which causes a significant work function shift. The presence of a dipole moment generates a weak electric field, which in turn alters the local potential distribution on the material surface; this electric field induces a downward shift in the surface vacuum level (the vacuum level acts as the energy reference for electrons to escape from the material surface), thereby effectively reducing the work function of the system [60]. Simply put, the formation of a surface dipole moment makes it easier for electrons to escape from the material surface. After high-density CO adsorption, the work function of the system shows a distinct decreasing trend with a variation of 0.44 eV, presenting a negative work function change. This phenomenon indicates that the reduction in work function may stem from the downward shift in the vacuum level caused by the surface dipole moment. Notably, the ΔΦ value of 0.44 eV for FePt–N_4_–C(III) is comparable to or even higher than many reported 2D sensing materials systems, such as NO_2_/WS_2_-based (+0.46 eV) [61], NO_2_/Sc_2_CF_2_ (+0.46 eV) [62], CO/Au–Ti_3_C_2_ (+0.07 eV) [56], H_2_O/SnO_2_@graphene (+0.180 eV) [57]. The consistency between our computed ΔΦ and these experimentally validated or theoretically predicted values further corroborates the strong surface electronic response of FePt–N_4_–C(III) and position.

In general, the macroscopic electrical conductivity (σ) of a sensing material changes significantly after adsorbing target gas molecules. This variation is closely related to the evolution of the electronic structure of the two-dimensional sensing system in this study. The relationship between electrical conductivity and bandgap follows: σ ∝ exp(−*E*_g_/2*K*_B_*T*). where *E*_g_, *K*_B_, and *T* are the bandgap, Boltzmann constant, and temperature, respectively. At a constant temperature, the bandgap *E*_g_ is the decisive factor regulating the electrical conductivity, and its small variation can induce a drastic fluctuation in conductivity through the exponential mechanism. Therefore, the bandgap change induced by gas adsorption can be directly converted into a strong and detectable electrical signal, thereby realizing efficient detection of the target gas. The bandgap of FePt–N_4_–C(III) is 0.019 eV, which increases significantly to 0.395 eV after CO adsorption. This obvious bandgap variation indicates that CO adsorption can induce a dramatic change in the electrical conductivity of the system, suggesting that FePt–N_4_–C(III) exhibits high gas-sensing sensitivity toward CO molecules. This conclusion is consistent with the work function analysis.

**Figure 8:**
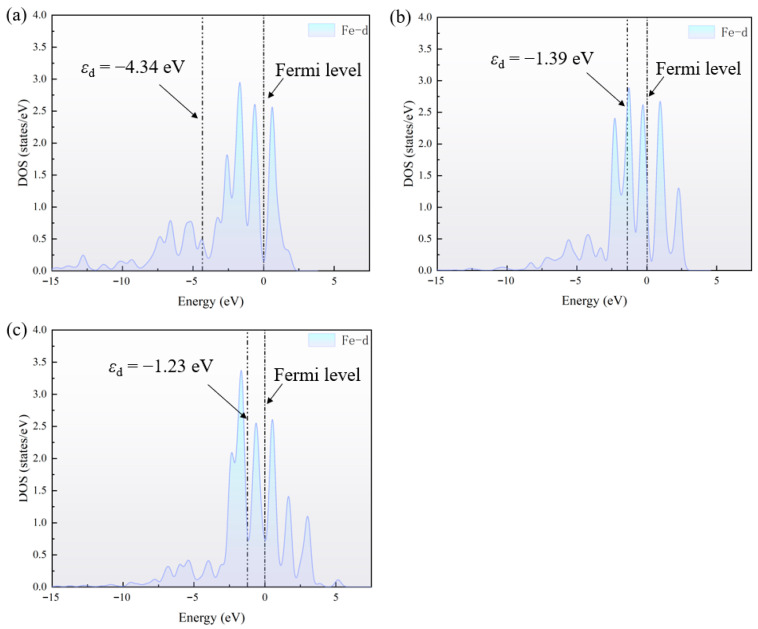
DOS of (**a**) FePt–N4–C(I), (**b**) FePt–N4–C(II), and (**c**) FePt–N4–C(III). The d-band center (*ε*_d_) of the Fe site, extracted from projected DOS analysis, exhibits a monotonic upshift with increasing site density, correlating with the enhanced CO adsorption strength.

The energy difference between the *ε*_d_ and the Fermi level can reflect the bonding strength between the adsorbate and the material surface; the closer the *ε*_d_ is to the Fermi level, the stronger the adsorption; otherwise, the adsorption is weaker. It can be seen that the *ε*_d_ of FePt–N_4_–C(I) is further away from the Fermi level than FePt–N_4_–C(II) and FePt–N_4_–C(III), indicating that FePt–N_4_–C(I) has weaker adsorption of CO than FePt–N_4_–C(II) and FePt–N_4_–C(III). This result is consistent with the calculation of *E*_ads_. As shown in Figure 8, the *ε*_d_ values for FePt–N_4_–C(I), (II), and (III) are −1.82, −1.73, and −1.58 eV, respectively. A clear monotonic upshift of the d-band center is observed with increasing active-site density. This trend directly parallels the adsorption energy trend on the Fe site (|*E*_ads_| increases from 1.36 to 1.49 to 1.52 eV from type I to III), providing a consistent electronic rationale: higher site density induces stronger Fe–d band coupling, shifting the d-band upward and enhancing the back-donation into the CO 2π* orbital. It is well established that Pt generally exhibits a lower-lying d-band center relative to the Fermi level compared to Fe [63]. This inherent electronic difference qualitatively explains the intrinsically weaker CO binding on Pt sites observed in our calculations, as a lower d-band center reduces the extent of π back-donation into the CO 2π* orbital.

### 3.4. Recovery Time

Recoverability—the ability of adsorbed gas molecules to desorb from the sensing surface—is a critical parameter for practical, reusable gas sensors. The recovery time (τ), estimated via transition state theory (Equation (3)), represents the average time required for sensor regeneration and serves as a key metric for desorption kinetics. Generally, weaker adsorption (less negative *E*_ads_) leads to shorter recovery times. However, excessively rapid desorption may compromise sensitivity, especially for trace gas detection. Thus, an optimal sensing material must strike a balance between sufficient adsorption strength and feasible recovery. Table 2 summarizes the calculated recovery times of CO on all TM_2_–N_4_–C configurations at three representative temperatures (298, 398, and 498 K).

Notably, within the FePt series, the recovery time exhibits a dramatic and monotonic increase with site density at room temperature: from ~10^11^ s (type I) to ~10^13^ s (type II and type III)—so higher density is not automatically better for practical sensing. In stark contrast, all PtPt–N_4_–C configurations show ultrafast recovery (τ ~10^−9^–10^−8^ s at 298 K) due to physisorption (*E*_ads_ > −0.3 eV). While this implies excellent reversibility, the concomitantly weak response signal renders them ineffective for reliable CO detection, especially at low concentrations. In addition, at 298 K, the complete CO desorption recovery times for Fe-containing systems under three different active site densities all exceed 10^11^ s. Such prolonged recovery times restrict their application as reusable gas sensors, rendering them suboptimal for sensing purposes. However, their strong CO adsorption capacity lends them potential value in CO capture or scavenging applications.

#### Temperature Effect and Density Trend

Temperature dramatically accelerates desorption. For all chemisorbing systems (FeFe and FePt), increasing the operating temperature from 298 K to 498 K reduces the recovery time by 6–14 orders of magnitude (Table 2). This exponential sensitivity to temperature is consistent with the Arrhenius-like dependence in Equation (3) and offers a practical pathway to tune the sensor between “capture mode” (room temperature, long τ) and “detection mode” (elevated temperature, short τ).

Site density exhibits contrasting trends for homonuclear and heteronuclear systems. For FeFe–N_4_–C, the recovery time increases monotonically with increasing site density at all temperatures. This trend is consistent with the monotonic increase in CO adsorption strength with density for FeFe systems (Section 3.2): stronger chemisorption at higher density (|*E*_ads_| increases from 1.37 to 1.46 eV) leads to longer desorption times, as dictated by the exponential dependence of τ on *E*_ads_ in Equation (3). The enhanced binding at closer Fe–Fe proximity likely originates from the upshifted d-band center and strengthened Fe–CO π back-donation, rather than from competitive or weakening effects.

For FePt–N_4_–C, the recovery time increases monotonically with site density. At 498 K, the recovery time follows: τ_III_ > τ_II_ > τ_I_. Notably, the low-density FePt–N_4_–C(I) achieves a recovery time of 10^2^ s at 498 K, which is more than nine orders of magnitude shorter than its room-temperature value (10^11^ s), while still retaining strong chemisorption at RT (*E*_ads_ = −1.36 eV). This significant acceleration of desorption at elevated temperature highlights the potential of temperature-modulated operation for FePt–N_4_–C(I), although its recovery time at 498 K remains too long for real-time sensing applications. The even longer recovery times of the medium- and high-density configurations at 498 K (~10^4^ s) further underscore the strong influence of site density on desorption kinetics.

Heteronuclear synergy can be revisited from a kinetic perspective. The distinctive behavior of FePt–N_4_–C(I)—moderate adsorption strength (*E*_ads_ = −1.36 eV) coupled with the fastest high-temperature desorption (τ = 65 s at 498 K) among the FePt series—arises from a unique interplay between its electronic structure and geometric spacing. In the FePt–N_4_–C(I) system, the d-band center sits at −1.82 eV. Because this upshift is less pronounced than in denser configurations (Section 3.3), the resulting adsorption energy naturally remains modest. However, the critical advantage here is the expansive 12.7 Å inter-site spacing. This wide physical separation effectively shuts down direct metal–metal interference. By alleviating such kinetic trapping, the adsorbed CO can desorb much more freely once thermally activated. What we observe is a clear decoupling of thermodynamics and kinetics: the local d-band position firmly dictates the initial binding strength, while the spatial isolation of the active sites ultimately lowers the barrier for thermal escape.

**Figure 9:**
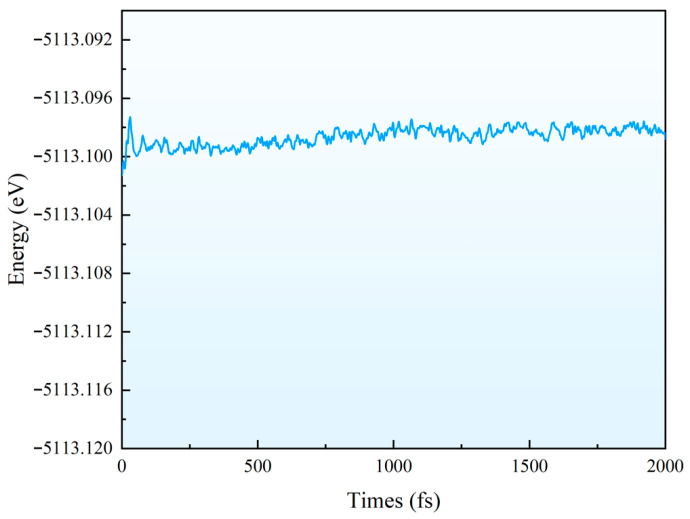
FPMD of FePt–N_4_–C(I) at 600 K.

Furthermore, we calculate the recovery time of FePt–N_4_–C(I) at 600 K to be 0.29 s. This once again confirms that temperature plays a crucial role in deciding the recovery time. We adopt the calculation method of first-principles molecular dynamics (FPMD) to analyze the stability of FePt–N_4_–C(I) at 600 K. The FPMD simulation shows that the calculated results are oscillating near the equilibrium state, indicating FePt–N_4_–C(I) is stable at 600 K, as shown in Figure 9. The structural changes before and after FPMD simulation are also shown. Obviously, the results show that the structure of FePt–N_4_–C(I) does not undergo large deformation, indicating that it has good stability at 600 K.

### 3.5. Spin-Polarized Computations

Ferromagnetism can significantly affect CO adsorption by inducing obvious spin polarization on the active sites [38,39,40,41]. Therefore, FePt–N_4_–C(I), FePt–N_4_–C(II), and FePt–N_4_–C(III) were selected as representative models to systematically investigate the effect of spin states on their sensing performance. As mentioned previously, in the FePt system, the Fe site serves as the primary adsorption center for CO. This section focuses on CO adsorption at the Fe site. Previous studies have shown that Fe(II) ions can exist in three spin states, namely S = 0, 1, and 2 [37]. Since the adsorption behavior at S = 0 was systematically analyzed in the previous section, we further calculated and compared the energies of the S = 1 and S = 2 states. Among all three FePt–N_4_–C configurations, the S = 1 spin state is thermodynamically superior to S = 2, with a total energy lower by 0.4–0.5 eV (Table A1, Appendix A). Therefore, the S = 1 state is the focus of our analysis.

**Figure 10:**
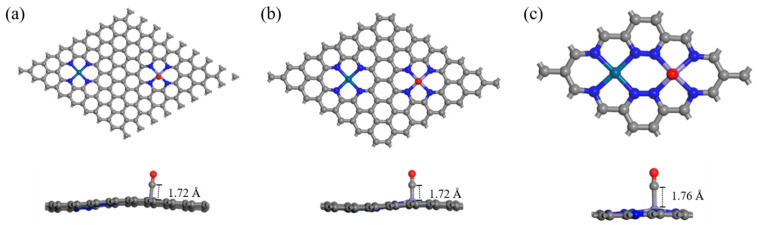
Optimal adsorption structures of CO adsorbed on (**a**) FePt–N_4_–C(I), (**b**) FePt–N_4_–C(II), and (**c**) FePt–N_4_–C(III) at S = 1. In all three configurations, CO prefers C-end atop adsorption on the Fe center and exhibits a short, near-linear geometry (Fe–C–O angle ≈ 175–180°) with Fe–C bond lengths of ~1.72 Å (I), ~1.72 Å (II), and ~1.76 Å (III), indicating chemisorption; increasing active-site density from I to III slightly elongates the Fe–C bond while the overall adsorption motif remains unchanged.

The calculated CO adsorption energies (*E*_ads_) on FePt–N_4_–C(I), (II), and (III) at the S = 1 state are −1.61, −1.84, and −1.63 eV, respectively (Figure 10). Compared to the non-spin-polarized (S = 0) results (−1.36, −1.49, and −1.52 eV), two critical observations emerge: (i) Spin polarization generally enhances CO chemisorption on Fe centers, with *E*_ads_ becoming more negative. This enhancement can be attributed to the spin-polarized d-band upshift and increased density of unpaired d-electrons available for back-donation into the CO 2π* orbital. (ii) Strikingly, the density-dependent trend is qualitatively reversed. At S = 0, adsorption strength increases monotonically with site density (III > II > I). At S = 1, the trend becomes non-monotonic, with type II exhibiting the strongest adsorption (−1.84 eV), followed by type I (−1.61 eV), while type III shows weakened adsorption (−1.63 eV). This reversal indicates that the impact of spin polarization is itself density-dependent: at high site density (III), the Fe center is more susceptible to spin-state switching that reduces its affinity for CO; at medium/high density (II, III), intersite coupling stabilizes a high-spin configuration that is more reactive.

Geometrically, CO remains adsorbed in a near-linear, upright configuration on Fe sites in all S = 1 cases (Fe–C–O angle > 170°), similar to the S = 0 geometry for types I and II. However, for FePt–N_4_–C(III), the adsorption distance increases slightly (from 1.74 Å to 1.79 Å), consistent with its weakened binding energy.

**Figure A3:**
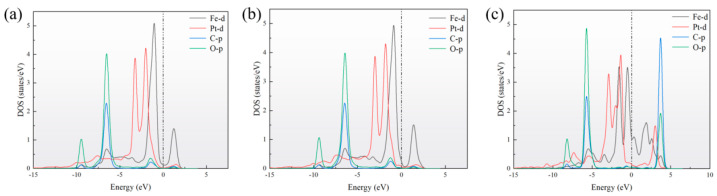
Spin-polarized projected density of states (PDOS) at S = 1 for (**a**) FePt–N4–C(I), (**b**) FePt–N4–C(II), and (**c**) FePt–N4–C(III).

**Figure A4:**
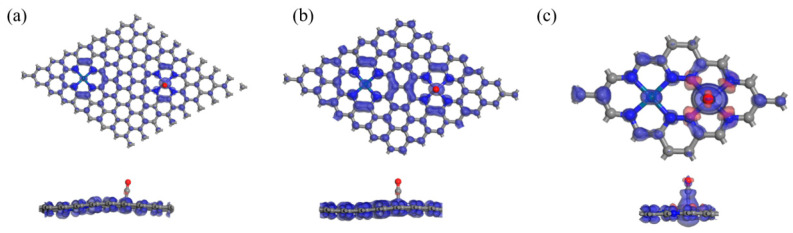
Spin density distribution (Δρ = ρ↑ − ρ↓) for (**a**) FePt–N_4_–C(I),(**b**) FePt–N_4_–C(II), and (**c**) FePt–N_4_–C(III) upon CO adsorption. Blue and red isosurfaces represent spin-up and spin-down components, respectively. Top and side views are shown.

To elucidate the electronic mechanisms underlying the spin-state-dependent and density-dependent CO adsorption trends, we analyzed the spin-polarized pDOS (Figure A3, Appendix A), total magnetic moments, and spin density distributions for the three FePt–N_4_–C configurations at the S = 1 state (Figure A4, Appendix A).

The involvement of Pt is consistent with a modulation of the Fe electronic states through Fe–Pt coupling, as reflected by the concurrent Fe-d and Pt-d features in the PDOS. For the high-density FePt–N_4_–C(III), the very short site–site separation (~4.2 Å) is expected to enhance inter-site electronic coupling, which is consistent with the substantial Fe-d states near *E_F_* in the PDOS (Figure A3c). In configuration III, the pronounced Fe-d weight near *E_F_* (Figure A3c) indicates a more metallic electronic character and stronger inter-site coupling compared with configurations I and II, which show a depletion of states near *E_F_* (Figure A1a,b). Such electronic structure differences are expected to modify the adsorption response under spin polarization. The spin density remains appreciably localized on Fe, but the competitive inter-site electronic effects dominate, leading to an opposite trend of spin polarization seen in the lower-density configurations.

Before CO adsorption, the total magnetic moments of FePt–N_4_–C(I), FePt–N_4_–C(II), and FePt–N_4_–C(III) are all approximately 2.0 *μ*_B_, with negligible magnetic moments on the Pt and N atoms. This indicates that the spin density was highly localized on the Fe center. Upon CO adsorption, while the total magnetic moment of the system remained unchanged across all three configurations, the local magnetic moment of the Fe center underwent a drastic reduction, decreasing from an initial ~2.0 *μ*_B_ to 0.172, 0.139, and 0.492 *μ*_B_, respectively. This phenomenon reveals that the adsorption process involves not only significant charge transfer but also a profound redistribution of spin density, wherein the spin density originally localized on the Fe extensively delocalizes onto the CO ligands and the surrounding support. Notably, although configuration FePt–N_4_–C(III) also exhibits spin delocalization, its Fe center retains a relatively high local magnetic moment, approximately three times that of FePt–N_4_–C(I) and FePt–N_4_–C(II). This striking discrepancy strongly implies the presence of competitive inter-site electronic interactions in FePt–N_4_–C(III). We propose that, within the specific geometric environment of FePt–N_4_–C(III), the orbital hybridization between the Fe atom and the adjacent Pt atom competes with the Fe–CO adsorption bond. This competition attenuates the strength of π-backdonation from Fe to CO, thereby hindering complete spin pairing and delocalization, and allowing the Fe center to retain a larger fraction of unpaired electrons.

Overall, the PDOS indicates a density-dependent change in the electronic structure: configurations I and II exhibit a pronounced depletion of states near *E_F_*, whereas configuration III shows substantial Fe-d states at *E_F_*, suggesting enhanced inter-site coupling and a more metallic character at high density. These electronic-structure differences provide a plausible basis for a density-dependent adsorption response under spin polarization and highlight that spin effects should be discussed together with geometric site density.

## 4. Conclusions

In this work, we have systematically investigated, via first-principles DFT calculations, how the density of bimetallic active sites (FeFe, PtPt, and FePt) anchored on N-doped graphene governs the structural, electronic, and CO sensing properties of TM_2_–N_4_–C systems. By integrating thermodynamic, electronic, kinetic, and spin-polarized analyses, we establish a comprehensive mechanistic picture with the following key findings. All TM_2_–N_4_–C configurations are thermodynamically stable (*E*_f_ < 0). For a given metal composition, heteronuclear FePt consistently outperforms homonuclear counterparts. As the density increases, the bandgap exhibits a non-monotonic evolution (0.43 eV → 0.59 eV → 0.019 eV). This phenomenon is due to the isolated behavior at low density, the band gap broadening caused by overlapping wave functions at medium density, and the near band gap closure dominated by orbital hybridization at high density. CO undergoes physical adsorption at Pt sites and chemical adsorption at Fe sites. In homonuclear FeFe systems, adsorption strength increases monotonically with site density (|*E*_ads_|: 1.37 → 1.41 → 1.46 eV). In heteronuclear FePt systems, adsorption strength also increases with density (1.36 → 1.49 → 1.52 eV). At different densities, FePt heteronuclear cells exhibit stronger adsorption capacity than mononuclear cells at all densities, demonstrating a significant synergistic effect. These trends are quantitatively rationalized by the monotonic upshift of the Fe d-band center with increasing density (−1.82 → −1.73 → −1.58 eV), which enhances π back-donation. Work function calculations reveal that CO adsorption significantly alters the surface electronic properties of TM_2_–N_4_–C.

Moreover, the recovery time of the sensor can be significantly adjusted by increasing the density of active centers and using the synergistic effect of heteronuclear bimetallic system. At room temperature, all Fe-containing systems exhibit ultra-long τ (>10^5^ s), suitable for CO capture/removal rather than reversible sensing. PtPt systems desorb instantaneously (τ ~ns) but lack sufficient response. The low-density FePt–N_4_–C(I) achieves a recovery time of 65 s at 498 K. Although 65 s remains too long for real-time applications, it highlights the potential of temperature-modulated operation. Spin-polarized calculations reveal that the S = 1 state is thermodynamically preferred for all FePt systems, with adsorption energies of −1.61 eV (I), −1.84 eV (II), and −1.63 eV (III). The density trend no longer follows the original order. Type II now binds most strongly, while type III shows a substantial loss of binding strength. This reversal is driven by the density-dependent evolution of the electronic structure. At low and medium densities, the states near the Fermi level (*E_F_*) are significantly depleted. In this regime, spin delocalizes onto the Pt sites, which enhances the adsorption response. At high densities, however, the shorter site-to-site separation (~4.2 Å) strengthens inter-site electronic coupling. This drives substantial Fe-d weight near *E_F_*, giving the system a noticeably more metallic character. These competitive inter-site effects eventually override the spin-driven gains, resulting in weakened adsorption. Our findings translate into three practical guidelines for sensor design: (i) For reversible, temperature-modulated sensing, the low-density (FePt–N_4_–C(I)) configuration is ideal, as it neatly decouples moderate adsorption from faster high-T desorption; (ii) when trace CO must be captured with absolute sensitivity, the medium-density FePt–N_4_–C(II) framework is required, as its high-spin (S = 1) ground state anchors the molecule deeply (*E*_ads_ = −1.84 eV) and deliberately forfeits reversibility; (iii) for work-function-based sensors, the high-density FePt–N_4_–C(III) configuration is highly suitable. Its near-gap closure promotes interfacial charge transfer, resulting in a distinct work function change (ΔΦ = +0.44 eV). This work demonstrates that active-site density is not merely a structural parameter but a powerful electronic and kinetic tuning knob that, when synergistically combined with heteronuclear composition and spin-state control, enables rational design of graphene-based CO sensors with performance tailored to specific application scenarios.

## Figures and Tables

**Table 1:** Distance and adsorption energies of CO.

	System	*D* (Å)	*E*_ads_ (eV)
I	FeFe–N_4_–C	2.01	−1.37
	PtPt–N_4_–C	3.32	−0.18
	FePt–N_4_–C	1.71	−1.36
II	FeFe–N_4_–C	1.72	−1.41
	PtPt–N_4_–C	3.65	−0.22
	FePt–N_4_–C	1.72	−1.49
III	FeFe–N_4_–C	1.73	−1.46
	PtPt–N_4_–C	3.06	−0.28
	FePt–N_4_–C	1.74	−1.52

**Table 2:** Recovery times in seconds of CO adsorbed on TM_2_–N_4_–C at 298, 398, and 498 K.

		298 K	398 K	498 K
I	FeFe–N_4_–C	1.69 × 10^11^	2.46 × 10^5^	79.42
	PtPt–N_4_–C	1.27 × 10^−9^	2.05 × 10^−10^	7.05 × 10^−11^
	FePt–N_4_–C	1.21 × 10^11^	1.93 × 10^5^	65.25
II	FeFe–N_4_–C	9.20 × 10^11^	8.76 × 10^5^	218.74
	PtPt–N_4_–C	4.46 × 10^−9^	5.40 × 10^−10^	1.52 × 10^−10^
	FePt–N_4_–C	2.16 × 10^13^	9.32 × 10^6^	1446.57
III	FeFe–N_4_–C	6.01 × 10^12^	3.57 × 10^6^	672.13
	PtPt–N_4_–C	5.08 × 10^−8^	3.33 × 10^−9^	6.54 × 10^−10^
	FePt–N_4_–C	5.47 × 10^13^	1.86 × 10^7^	2521.20

## Data Availability

The original contributions presented in this study are included in the article. Further inquiries can be directed to the corresponding author.

## Appendix A. Implications for Sensor Design

The spin-polarized calculations provide two important insights for the rational design of graphene-based CO sensors. First, they demonstrate that non-spin-polarized DFT systematically underestimates the CO adsorption strength on Fe centers in their preferred high-spin (S = 1) state for low- and medium-density configurations, with underestimations of 0.25 eV (type I) and 0.35 eV (type II). Accurate quantitative prediction of sensing performance for Fe-containing systems therefore requires explicit treatment of spin polarization. For the high-density configuration (type III), however, non-spin-polarized DFT overestimates the adsorption strength (by 0.42 eV), as strong inter-site d–d hybridization overrides spin-driven gains and leads to d-band downshift upon spin polarization.

Second, spin-state engineering emerges as a density-coupled tuning knob that can qualitatively alter the adsorption landscape. The optimal spin response is achieved at medium density (type II), where partial spin delocalization onto the adjacent Pt atom amplifies the d-band upshift and yields the strongest CO binding (−1.84 eV). If this S = 1 state can be stabilized experimentally (e.g., via ligand field design, support effects, or external magnetic field), it could be exploited for ultra-sensitive trace CO capture applications where maximum binding strength is desired, even at the cost of reversibility. In contrast, the low-density configuration (type I) exhibits a modest but still beneficial spin-induced enhancement (ΔE = 0.25 eV), and its faster high-temperature desorption kinetics (τ = 65 s at 498 K) may render it more suitable for reversible sensing under temperature-modulated operation. The high-density configuration (type III), where spin polarization actually weakens CO binding, is clearly unfavorable for either capture or sensing applications involving Fe centers.

In summary, spin polarization not only quantitatively modulates the adsorption energetics but also qualitatively reorders the density-dependent trends established in Section 3.2. A complete description—and rational design—of CO sensors based on Fe-containing bimetallic graphene must therefore account for both the active-site density and the accessible spin states, as their interplay determines the ultimate adsorption and desorption characteristics.

**Table A1:** Total energies of the three FePt–N_4_–C configurations calculated for the S = 1 and S = 2 spin states.

System	S = 1/eV	S = 2/eV	Energy Difference (S = 2 − S = 1)
FePt–N_4_–C(I)	−138,751.2063	−138,750.7844	+0.4219 eV
FePt–N_4_–C(II)	−107,660.5679	−107,660.1469	+0.4210 eV
FePt–N_4_–C(III)	−39,260.14843	−39,259.61845	+0.52998 eV
